# Structural and Functional Abnormalities in the Olfactory System of Fragile X Syndrome Models

**DOI:** 10.3389/fnmol.2019.00135

**Published:** 2019-05-28

**Authors:** Felipe Bodaleo, Carola Tapia-Monsalves, Christian Cea-Del Rio, Christian Gonzalez-Billault, Alexia Nunez-Parra

**Affiliations:** ^1^Laboratory of Cellular and Neuronal Dynamics, Department of Biology, Faculty of Science, Universidad de Chile, Santiago, Chile; ^2^Geroscience Center for Brain Health and Metabolism (GERO), Santiago, Chile; ^3^Biomedical Research Center, Universidad Autónoma de Chile, Santiago, Chile; ^4^Laboratory of Neurophysiopathology, Centro de Investigacion Biomedica y Aplicada (CIBAP), School of Medicine, Universidad de Santiago de Chile, Santiago, Chile; ^5^The Buck Institute for Research on Aging, Novato, CA, United States; ^6^Department of Biology, Faculty of Science, Universidad de Chile, Santiago, Chile; ^7^Cell Physiology Center, Universidad de Chile, Santiago, Chile

**Keywords:** olfactory coding, olfactory behavior, *Fmr1*-KO, FMRP, *dfmr1*, structural plasticity, excitation/inhibition balance

## Abstract

Fragile X Syndrome (FXS) is the most common inherited form of intellectual disability. It is produced by mutation of the *Fmr1* gene that encodes for the Fragile Mental Retardation Protein (FMRP), an important RNA-binding protein that regulates the expression of multiple proteins located in neuronal synapses. Individuals with FXS exhibit abnormal sensory information processing frequently leading to hypersensitivity across sensory modalities and consequently a wide array of behavioral symptoms. Insects and mammals engage primarily their sense of smell to create proper representations of the external world and guide adequate decision-making processes. This feature in combination with the exquisitely organized neuronal circuits found throughout the olfactory system (OS) and the wide expression of FMRP in brain regions that process olfactory information makes it an ideal model to study sensory alterations in FXS models. In the last decade several groups have taken advantage of these features and have used the OS of fruit fly and rodents to understand neuronal alteration giving rise to sensory perception issues. In this review article, we will discuss molecular, morphological and physiological aspects of the olfactory information processing in FXS models. We will highlight the decreased inhibitory/excitatory synaptic balance and the diminished synaptic plasticity found in this system resulting in behavioral alteration of individuals in the presence of odorant stimuli.

## Introduction

Fragile X syndrome (FXS) is one of the most common causes of inherited intellectual disability and the most common monogenetic cause of autism. It is estimated that the syndrome prevalence is 1 in 5,000–7,000 in males, while in females is 1 in 8,000–11,000. It is produced by the repeat expansion of the CGG trinucleotide in the promoter region of the human *FMR1* gene located on chromosome X which leads to hypermethylation and transcriptional silencing of the gen. Individuals with more than 200 CGG repetitions exhibit the full mutation and FXS (Hagerman et al., 2017; Nelson et al., 2017; Sherman and Hunter, 2017).

The Fragile Mental Retardation Protein (FMRP) is a selective RNA-binding protein that regulates the transcription of 4% of the total proteins found in the mammalian brain (Ashley et al., 1993), where its primary function is to repress local protein translation of specific mRNAs at dendrites in an activity-dependent manner, down-regulating the synthesis of proteins involved in synaptic plasticity and function (Brown et al., 2001; Darnell and Klann, 2013; Sidorov et al., 2013; Suhl and Hoeffer, 2017; Bagni and Zukin, 2019). Recently, however, evidence supports additional roles for FMRP, positively regulating mRNA translations and modulating protein activity or molecules stability by direct interaction (Davis and Broadie, 2017; Bagni and Zukin, 2019). It is important to highlight that FMRP interacts with a broad range of coding mRNAs, regulating its translation in both murine and human brain (Ascano et al., 2012; Maurin et al., 2018) and in adult neuronal stem cells (Liu et al., 2018). Moreover, FMRP binds to the coding region of transcripts encoding both pre- and post-synaptic proteins (Darnell et al., 2011), hence playing a major role in learning, memory, adaptation and sensory perception. FXS individual’s symptoms include learning disabilities, attention deficit and behavioral and social alterations such as hyperactivity, impaired social communication, hyperarousal and extreme sensitivity to sensory stimuli, among others. Some of all abnormal social behavior in FXS might be secondary to inappropriate filtering to daily life sensory stimulus and a consequent altered sensitivity across sensory modalities including olfaction (Hagerman et al., 1996; Miller et al., 1999; Christie et al., 2009; Rotschafer and Razak, 2013; Arnett et al., 2014; Juczewski et al., 2016; Goel et al., 2018), yet compared to cognitive and social functioning how sensory information is processed in FXS has been largely understudied.

To gain insights into the pathology, physiology and molecular processes of FXS, researchers have predominately used rodents and *Drosophila melanogaster* as experimental animal models. These models have been genetically manipulated to emulate the genotype of FXS, knocking out or down the *Fmr1* gene in mice and *Drosophila*. In both models, the olfactory system (OS) is the most conserved sensory system and critical for the species survival and reproduction, an ideal candidate to study sensory information processing issues found in the absence of FMRP. Moreover, the OS has several traits that make it a highly attractive system to study the neuromorphological and neurophysiological aspects of sensory perception in FXS: (1) in humans, FMRP expression has been confirmed in olfactory neuroblasts, harvested through a nasal biopsy of control individuals and its absence corroborated in patients with FXS (Abrams et al., 1999). Similarly, in rodents and flies FMRP is expressed in high levels in the adult and developing rodent OS (Zhang et al., 2001; Schenck et al., 2002; Christie et al., 2009; Akins et al., 2012; Sudhakaran et al., 2014); (2) the OS has an exquisitely organized neuronal circuitry with a layered anatomy where the input and output information can be easily identified (Farbman, 1992; Murthy, 2011); (3) experimental models exhibit stereotyped olfactory-mediated behaviors, making it an ideal model to pair physiology with behavior, and (4) together with the hippocampus, the OS is the only brain region that exhibits adult neurogenesis in rodents (Lois and Alvarez-Buylla, 1993; Gheusi et al., 2012), which allows to study the dynamics of neuronal proliferation, migration, maturation, synaptic integration and ultimately experience-mediated plasticity in the post-natal brain.

In this review article, we will discuss molecular, morphological and physiological aspects of olfactory information processing in *Drosophila* and rodents in the absence of FMRP.

## Olfactory Discrimination in FXS Models

Mice where the *Fmr1* gene has been knocked out (*Fmr1*-KO; The Dutch-Belgian Fragile X Consortium et al., 1994) exhibit some phenotypic features similar to humans with FXS such as hypersensitivity, hyperactivity, diminished attentive capacity and anxiety (Kooy et al., 2017). FMRP is widely expressed in the rodent brain in the somatodendritic domain of virtually all neurons. In the OSs it is expressed throughout the brain regions that process olfactory information, from the peripheral olfactory sensory neurons (OSNs) in the Olfactory epithelium (OE), to all the neuronal types found in the olfactory bulb (OB) and olfactory cortex (OC, Box 1; Hinds et al., 1993; Christie et al., 2009; Akins et al., 2012; Brackett et al., 2013). FMRP is also localized at pre-synaptic terminals, in discrete granules called Fragile X Granules (FXG). FXG are structures that comprise proteins, ribosomes and mRNA and can be found only in a subset of brain regions including the axons of OSNs and in the glomeruli neuropil in the OB, suggesting that FMRP could not only have a post-synaptic role regulating post-synaptic protein translation but also be involved in plastic pre-synaptic olfactory processes (Christie et al., 2009; Akins et al., 2012; Korsak et al., 2017) as have been shown in hippocampal pyramidal neurons (Deng et al., 2013; Myrick et al., 2015). The ubiquitous expression of FMRP in the OS suggests that it might play a role in odorant sensing and processing, as well as in higher order bulbar and cortical computations such as olfactory discrimination, learning and memory. Indeed, some groups have reported that *Fmr1*-KO mice exhibit olfactory dysfunctions, such as a decreased olfactory sensitivity, which refers to the ability of the animal to detect an odorant, when tested in a spontaneous cross-habitation task (Schilit Nitenson et al., 2015). In this task, an animal is presented with an odor consecutively for brief periods of time to induce habituation, which is reflected in a progressive reduction of the time the animal spends investigating the sample. After the habituation period a novel odor, or the same odor at a different concentration, is presented. Failure to increase the investigation time indicates cross-habituation or incapacity to discriminate between the two odorants (Cleland et al., 2002). *Fmr1*-KO, albeit they are able to discriminate odorants, increase the investigation time to a sample one log of concentration higher than WT controls (Schilit Nitenson et al., 2015), reflecting a decreased sensitivity for processing olfactory information. *Fmr1*-KO mice olfactory sensitivity has also been tested in a two-alternative olfactory discrimination forced choice task with contradictory results. In this task water deprived animals learn to poke with their nose one of two odor ports randomly delivering water as a reward. Under these circumstances, there is not any difference in olfactory sensitivity between *Fmr1*-KO and WT. The difference between these two studies could arise on the difference between the two behavioral paradigms chosen. The two-alternative forced choice, as opposed to the cross-habituation task, requires operant conditioning and the consolidation of a stimulus-reward association. This learning process engages top-down neuronal circuits such as cholinergic nuclei in the basal forebrain (Richardson and DeLong, 1991; Lin and Nicolelis, 2008) modifying how the bulbar neurons respond to a stimulus through context-dependent plasticity (Doucette and Restrepo, 2008), which may by itself regulated the perceptual threshold of an odor. Even though most *Fmr1*-KO mice displayed similar learning curves than control, during the shaping learning curves (where the animal learns the behavioral task itself) *Fmr1*-KO made more errors during the learning process with some *Fmr1*-KO not being able to reach criteria at all (Larson et al., 2008), emulating the intellectual deficits found in the majority of individuals with FXS (Hall et al., 2008). Thus, depending on the environmental conditions and decision-making requirements bottom up and top-down processes that regulate olfactory information computations could be altered in *Fmr1-KO*.

Box 1 **Olfactory System in Rodents and *Drosophila***

**Figure 1:**
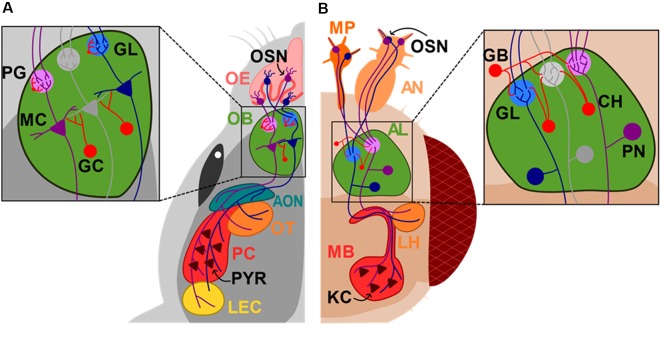
Diagram of the mouse and *Drosophila* olfactory systems. Sensory neurons (OSN) in the nose or antennas (AN) and maxillary palp (MP) project to the superior centers of olfactory sensory processing, olfactory bulb (OB) and antennal lobe (AL) in mice **(A)** and *Drosophila* **(B)**, respectively. Mitral cells (MC) send their axons directly to the accessory olfactory nucleus (AON), olfactory tubercle (OT), pyriform cortex (PC) and lateral entorhinal cortex (LEC), while projection neurons (PN) project to the mushroom body (MB) and lateral horns (LH). Olfactory epithelium (OE); glomerulus layer (GL); periglomerular cell (PG); granule cell (GC); GABAergic neurons (GB); cholinergic neurons (CH); kenyon cells (KCs). Neurons that are synaptically connected are depicted in the same colors.

Odorant molecules enter the nose through inhaled air and interact with olfactory receptors of sensory neurons (OSN) located in the nasal olfactory epithelium. To process the diverse and vast number of odors found in the environment, a combinatorial approach is used by the olfactory system. From a ~1,000 olfactory receptors (OR) found in rodents, each OSN in the olfactory epithelium expresses only one of them (Buck and Axel, 1991). An odor can activate different types of OR and each of these OR gets activated by different odorants exhibiting different tuning properties (Araneda et al., 2004). This property of the OR will produce a distributed pattern of activation of OSNs for each odor mixture. Interestingly, olfactory neurons expressing the same receptor project to only one or two glomeruli in the olfactory bulb (OB; Mombaerts et al., 1996), creating a two-dimensional distributed map of odor information across the glomerular layer (Mori et al., 1999). Mitral cells (MC) in the OB receive the upcoming information by extending their apical dendrite to only one glomerulus maintaining this map downstream and projecting their axon to pyramidal cells (Pyr) in the olfactory cortex (OC). Olfactory information is therefore coded in the OB by changes in MC activity that creates a spatio-temporal code, information that is later integrated and ultimately decoded by the cortex (Figure 1A). In flies, similarly, OSNs from the antenna and maxillary palp also express a single OR and project to a single glomerulus in the antennal lobe (AL) where they synapse onto the projection neurons (PN). PN also extend their dendrite to only one glomerulus, analogous to the rodent OB and MCs. PN, then send their axons to the Kenyon cells (KCs) in the mushroom body (MB), the learning and memory center of the fly and to the lateral horn (LH) in the protocerebrum (Su et al., 2009; Semaniuk, 2015; Figure 1B). Importantly, in both species, inhibitory neurons strictly regulate olfactory processing. In mice, periglomerular neurons (PG) in the glomerulus regulate the influx of information into the brain, while granule cells (GCs) regulate the efflux of information to the OC by making inhibitory synaptic contacts onto the dendrites of MCs. In *Drosophila*, GABAergic local neurons inhibit the pre-synaptic activity at the axon terminals of OSNs and excitatory cholinergic neurons mediate interglomerular excitation.

Impaired olfactory performance has also been observed in the fly model of FXS (*dfmr1*) where the absence of dFMRP (the human homolog of FMRP) resulted in reduced olfactory attraction and aversion. In the behavioral experiment, starved flies where presented with an attractive (ethyl acetate) or aversive (benzaldehyde) odorant in an arena with two chambers. The number of flies in the odorized and non-odorized section of a behavioral arena was then counted. They found that *dfmr1*^−^ flies spent less time exploring the quadrant with the attractive odor and that they were less repelled with the aversive odor compared to controls. The same behavioral phenotype was observed when dFMRP was selectively downregulated in the antennal lobe (AL) projection neurons (Franco et al., 2017), suggesting that the behavioral alteration could originate in a somehow dysregulated olfactory projection neuron (PN, Box 1) activity. Flies *dfmr1*^3^ heterozygous also show defects in the olfactory associative learning test (OAT) or negatively reinforced paradigm, where an odor (CS) is delivered to a chamber in parallel with foot shocks and later the number of flies that prefer a chamber with the CS are counted (Kanellopoulos et al., 2012). These results suggest that FMRP expression is required to adequately process olfactory information and generate context-dependent memories. The role that FMRP plays in olfactory sensing (or sensory processing in general) is not yet understood, but some evidence started to shed light on its function regulating structural plasticity and neuronal excitability.

## Anatomical Alterations in the Olfactory Systems of FXS Models

In agreement with a role that FMRP plays regulating neuronal branching (Morales et al., 2002; Galvez et al., 2005), anatomical defects have been found in the olfactory system of FXS models. For instance, mitral cells (MCs) from *Fmr1*-KO exhibit altered architecture when compared to WT controls. As described in Box 1, MCs usually project only one dendrite to a unique glomerulus (GL; Malun and Brunjes, 1996) transmitting information in parallel columns downstream the OB and into the olfactory cortex (OC). *Fmr1*-KO OB, however, has on average two apical dendrites per MC (Galvez et al., 2005). Whether the apical dendrites project to one or multiple GL in the *Fmr1*-KO has not been explored yet, but it can be hypothesized that this aberrant morphology would distort olfactory processing. If MCs project to multiple GL, a single MC will be activated by different types of OSNs expressing different Olfactory receptors (ORs), forcing the system to an early integration of information that would otherwise had occurred in the OC. On the other hand, in the case that both apical dendrites project to a single GL, the same glutamatergic release from the OSN will have an augmented effect in the MC and produce an artificially elevated perception of the stimulus as has been described in mice auditory and somatosensory cortex (Arnett et al., 2014) and in humans with FXS (Miller et al., 1999; Christie et al., 2009).

Structural deficits have also been found in the olfactory learning and memory center of the *dfmr1*^−^ flies. Specifically, Kenyon cells (KCs) in the mushroom body (MB) exhibit defects in axonal outputs and dendritic arborization (Pan et al., 2004, 2008; Doll and Broadie, 2015), while PN neurons show reduced neuronal branching, enlargement of the synaptic boutons and reduced connectivity with the post-synaptic KC (Doll et al., 2017). Structural abnormalities have also been observed in GABAergic neurons in flies lacking FMRP, displaying morphological alteration during development with early underdevelopment and later overcompensation (Gatto et al., 2014).

One of the most relevant histological features in neurons of individuals with FXS is the increased abundance of immature and elongated dendritic spines (Altman and Das, 1965; Comery et al., 1997; He and Portera-Cailliau, 2013). Inadequate size and morphology of spines are linked to altered neuronal connectivity, synaptic function and synaptic plasticity (Sala and Segal, 2014) suggesting an underlying role of spine abnormality in some of the symptoms observed in FXS. The classic methodological approximation used to measure spine density and morphology had been performed mostly in the postnatal brain of whole *Fmr1*-KO models, where the effect of FMRP absence in single neurons cannot be dissected from the potential large-scale synaptic effect of knocking down this protein in the whole system (He and Portera-Cailliau, 2013). To solve this problem, the rodent olfactory system exhibits a unique feature, only shared with the hippocampus: the ability of inhibitory neurons—granule cells (GCs) and periglomerular cells (PGs) to proliferate in the postnatal brain. Adult neurogenesis is a widespread process occurring in several organisms from insects to rodents, but it is very limited in *Drosophila* (von Trotha et al., 2009; Simões and Rhiner, 2017) subscribing scarcely only to the optical lobes (Fernández-Hernández et al., 2013). Rodent adult-born neurons originate from progenitor cells located in the subventricular zone (SVZ) of the brain, from where they migrate for about 2 mm to the OB and become functionally integrated within the OB network in a process that takes between 21 and 30 days (Altman and Das, 1965; Lois and Alvarez-Buylla, 1993, 1994). This spatial segregation allows for genetic manipulations to be performed exclusively in the neuronal progenitors and to study the effects of those manipulations at later time points in the OB after the neurons have migrated. Adult-generated GC, in which the *Fmr1* gene was knocked-down by injecting RNA-interference in SVZ, had denser and longer dendritic spines compared to control when evaluated 21 days post injection (d.p.i; Scotto-Lomassese et al., 2011), once the neurons have already reached the OB (Petreanu and Alvarez-Buylla, 2002). Healthy adult-generated GCs when are functionally integrated into the OB neuronal network (Bardy et al., 2010) form glutamatergic synapses with the lateral dendrites of the MCs (Belluzzi et al., 2003). Interestingly, knockdown *Fmr1* GC had more mature glutamatergic synaptic sites and accordingly received more glutamatergic inputs than control GCs (Scotto-Lomassese et al., 2011), which recapitulates the hyperexcitability phenotype found in other brain regions in FXS (Contractor et al., 2015; Ethridge et al., 2017). Interestingly, at 28 d.p.i, when new born GCs are fully mature, the number of dendritic spines in GC lacking FMRP was no different to control GCs (Scotto-Lomassese et al., 2011), suggesting that neurons without FMRP exhibited an accelerated rate of spinogenesis, that could homeostatically counterbalanced during development in an attempt to re-establish similar rates of connectivity in the FXS network. Taking together this evidence suggests that the absence of FMRP could interfere with normal neuronal architecture and synaptogenesis leading to olfactory dysfunctions in FXS.

## Structural Plasticity Alterations in the Olfactory System of FXS Models

It has been suggested that changes in the number or morphology of dendritic spines and dendritic arborizations, contributes in the regulation of the physiological changes of synaptic transmission underlying learning and memory (Lamprecht and LeDoux, 2004; Caroni et al., 2012). This process, known as structural plasticity, also occurs in healthy adult-born GCs in response to changes in the environment and olfactory input, but fail to occur in knockdown *Fmr1* GCs. For instance, in response to reduced sensory input a decrease in the complexity of the dendritic arborization is observed in WT GCs (Saghatelyan et al., 2005), process that does not occur in GCs lacking FMRP after animals were deprived of olfactory stimuli occluding one nostril unilaterally (Scotto-Lomassese et al., 2011). In addition, perceptual learning, the improved ability of the sensory system to discriminate stimuli based on experience, also induces profound morphological changes in rodent adult-born neurons. WT mice cannot naturally discriminate between the two perceptually similar odorants limonene+ (Lim+) and limonene− (Lim−), but when WT mice are pre-exposed to the odors for 10 days, they acquire the ability to discriminate between them. *Fmr1*-KO, however, cannot learn to discriminate between Lim+ and Lim−. This learning process in WT was accompanied with increase in the length and complexity of the dendritic branching and spine density in adult-born neurons, changes that were not observed in new neurons lacking FMRP (Daroles et al., 2016). In *Drosophila*, dFMRP has also been shown to play an important role in activity-dependent synaptic remodeling of the olfactory system, during the critical period. During this time, AL projection neurons (specifically AL-mPN2) of WT flies normally reduce their neuronal branch length after a passive exposure to pyrrolidine (Doll et al., 2017). Pyrrolidine is a natural aversive odorant to flies (Schlief and Wilson, 2007) that promotes structural plastic changes in AL-mPN2 neurons, which have a strong and highly specific response to it (Silbering et al., 2011). In the mutant *dfmr1*^−^ fly, the branch length is already diminished in basal conditions and the pyrrolidine-induced structural change reduction is not observed (Doll et al., 2017). Taken together, these results suggest that FMRP plays an important role in synapse formation and that the deficits in activity-dependent structural plasticity observed in GCs could mediate in part the cognitive defects found in the experimental FXS models.

At the neurophysiological level, long-term potentiation (LTP), a long-term change in synaptic strength involving morphological changes in dendritic spines (Nicoll, 2017) was studied *in vitro* in mice brain slices of the piriform cortex. The piriform cortex is a paleocortical three-layer structure that exhibits excitatory association fibers in layer 1b and is thought to play a major role in the formation of olfactory memories and olfactory discrimination (Bekkers and Suzuki, 2013). LTP induction by theta burst stimulation (TBS) in cortical layer 1b showed that *Fmr1*-KO mice had a substantial reduction of the LTP compared to controls in animals older than 6 months old. In flies, studies have shown that dFMRP directly interacts with Staufen and AGO1, two proteins that play a key role during long term memory (LTM) formation (Bolduc et al., 2008). These findings give a hint on the plastic processes that could be altered in the FXS, specifically in a brain region in charge of integrating sensory information and crucial to generate olfactory memories.

## Metabotropic Glutamate Receptor (mGluR) Theory and Olfaction

Recent studies have suggested that the *Fmr1*-KO mouse exhibit an imbalance between LTP and long-term depression (LTD; Contractor et al., 2015). LTD is another mechanism contributing to learning and memory and has been widely studied in FXS models giving rise to the “mGluR theory.” In this theory, the absence of FMRP has been associated to an increment of non-regulated protein synthesis mediated by the post-synaptic metabotropic glutamate receptor (mGluR). Once activated, mGluR stimulates the rapid translation of specific preexisting mRNAs in the dendritic spines which are involved in the internalization of AMPA receptors and in the generation of mGluR-mediated LTD in the synapse. This type of LTD occurs and is enhanced in* Fmr1-*KO mice, suggesting that FMRP is required to inhibit the translation of mRNAs involved in LTD stabilization (Snyder et al., 2001; Bear et al., 2004). Importantly, odorant-gated behavior could be rescued just by inhibiting mGluR activation in the conditional *Fmr1-*KO mouse, since injection of MPEP (a mGluR type 1 antagonist), rescued the learning deficits of these animal in a cross-habitation task and the dendritic arbor structural plasticity in bulbar adult-born GCs (Daroles et al., 2016). Similarly, *dfmr1*^3^ heterozygous flies fed with MPEP eliminated the behavioral alterations these flies exhibited in the olfactory associative test, where an odor is paired with an electric shock and the flies are later tasted for their preference to the odor (Kanellopoulos et al., 2012). Consistent with the ability of FMRP to act as negative regulator of mRNA translation (Laggerbauer et al., 2001; Mazroui et al., 2002), feeding *dfmr1*^−^ flies with low concentrations of the protein synthesis inhibitors cycloheximide and puromycin ameliorated olfactory-LTM deficits (Bolduc et al., 2008). Thus, modifying and decreasing protein synthesis directly by activating mGluR or unspecifically by protein synthesis inhibitors, could rescue olfactory behavioral phenotypes in FXS models.

## Inhibitory Circuit Dysfunction in the Olfactory System of FXS

Sensory processing alterations in FXS are believed to occur by an unregulated inhibitory/excitatory synaptic balance that could promote the observed hyperexcited state of neuronal circuit and ultimately underpin the increase number of seizures and hypersensitivities found in FXS (Contractor et al., 2015; Davis and Broadie, 2017). For instance, in the *Fmr1*-KO mouse, an hyperexcitable phenotype has been described in the auditory cortex (Rotschafer and Razak, 2013), where neurons exhibit an elevated responsiveness to auditory stimuli, which could lead to altered auditory processing and also a lower threshold for audiogenic seizures (Yan et al., 2005). Furthermore, in the somatosensory cortex of *Fmr1*-KO mice cellular deficits to adapt to repetitive whisker stimulation could account for sensory perception deficits and, more specifically, the tactile defensiveness phenotype seen in the syndrome (He et al., 2017). Moreover, *Fmr1*-KO mice exhibit larger sensory maps in the barrel and visual cortex impairing learning in a whisker- or visual-dependent behavioral task (Arnett et al., 2014).

In addition to the described expression of FMRP in excitatory neurons, FMRP is also present in inhibitory neurons (Olmos-Serrano et al., 2010; Gatto et al., 2014) suggesting that hyperexcitation is mediated in part by a faulty inhibitory system (Cea-Del Rio and Huntsman, 2014; Huntsman and Kooy, 2017).

In the olfactory system of the fly, albeit the general GABAergic neuron number is normal in *dfmr1*^−^, the GABA-synthetizing enzyme GAD is strongly reduced in the MB compared to WT individuals. GABA receptors are also downregulated in *dfmr1*^−^ AL PN and there is a decrease synaptic connectivity between interneurons and PN, suggesting the dFMRP might be required for proper inhibitory synaptic control (Gatto et al., 2014).

Appropriate inhibitory lateral interactions among GL on the AL are critical for odor information processing, especially for odor mixtures (Figure 1B, inset). In flies, lateral interactions narrows GL odor tuning (Olsen and Wilson, 2008) to enhance contrast and generate adequate olfactory representations downstream. The functional consequences of the faulty GABAergic system observed in *dfmr1*^−^ were studied measuring the changes in calcium dynamics using the fluorescent calcium sensor GCaMP3 in the GL. Indeed, when the fly glomeruli activity was measured in response to an odor, the response profile was broader in *dfmr1*^−^ flies, suggesting a decrease odor selectivity and contrast (Franco et al., 2017). In addition, GABAergic neurons from mutant flies innervating the MB have an augmented response to stimulation which was also observed by an increase in the fluorescence mediated by GCaMP3 (Gatto et al., 2014).

This evidence indicates that alterations in the inhibitory circuit is abnormal in FXS, which could in part promotes an excitatory/inhibitory imbalance and explain the altered olfactory information processing and olfactory-guided behavior observed in *dfmr1*^−^ flies.

## FMRP Interactors and Regulation of the FMRP Function in the Fly Olfactory System

One of the best-characterized function of FMRP is its role acting as a selective RNA-binding protein regulating the translation of multiple proteins related to synaptic physiology (Ashley et al., 1993). Important efforts have been made to study the FMRP molecular function underpinning the sensory alterations found in FXS using the olfactory system of *Drosophila* as the experimental model.

Futsch is a *Drosophila* protein orthologous to the mammalian microtubule-associated protein 1B (MAP1B) that plays crucial roles in dendritic and axonal growth during embryogenesis (Hummel et al., 2000; Roos et al., 2000) and regulates the synaptic microtubule cytoskeleton organization at the neuromuscular junction (NMJ; Bodaleo and Gonzalez-Billault, 2016). It has been shown that dFMRP physically interacts with *futsch* mRNA, acting as a negative translational regulator of Futsch (Zhang et al., 2001). Of note, both* futsch* overexpression and *dfmr1-*null mutants show a synaptic overgrowth phenotype at NMJ characterized by an increased number of synaptic boutons and enhanced levels of neurotransmission. Interestingly, double mutants of *dfmr1-* and *futsch* restore the altered synaptic phenotype to wild-type levels at NMJ (Zhang et al., 2001). In the fly adult olfactory system, *futsch* mutants show a progressive neuronal degeneration, accompanied by deficits in learning and olfactory memory. These detrimental phenotypes are partially suppressed by a *dfmr1* deletion (Bettencourt da Cruz et al., 2005). It has been shown that dFMRP is highly expressed in the larval MB (Schenck et al., 2002) and in PN (Bettencourt da Cruz et al., 2005), making it coherent that its misregulation could lead to defects in the olfactory sensory system. It is important to highlight that the mRNA of the mammal orthologous of *futsch*, *MAP1B*, and for *CAMKII* have been widely reported to interact with FMRP in the mammal brain (Davis and Broadie, 2017; Bagni and Zukin, 2019), opening the possibility that the impairment of these dFMRP-mRNA interactions may be recapitulated in the olfactory system of FXS mammal models.

Another mRNA regulated by dFMRP is *shrub* (human Chmp4). Shrub is a core member, the endosomal sorting complexes required for transport (ESCRT; Schmidt and Teis, 2012). Interestingly, dFMRP binds *shrub* mRNA to negatively regulate Shrub protein expression levels in whole brain lysates from newly eclosed animals during the disease state early-use critical period. Both dFMRP loss-of-function and Shrub overexpression increase PN innervation, PN synaptic endosomes and PN synaptic area (Vita and Broadie, 2017). Moreover, it has been suggested that Shrub controls neuronal morphogenesis in *Drosophila*, since Shrub-null animals display abnormal distribution of endosomal markers, and an altered axonal and dendritic branching pattern (Sweeney et al., 2006). These antecedents strongly suggest that membrane trafficking impairments at synapses could be a novel causative mechanism in the FXS disease (Vita and Broadie, 2017) which could negatively affect the structure and synaptic physiology in the olfactory network of FXS models.

In addition of acting as a translational regulator, FMRP functions themselves are regulated by its interaction with other proteins, post-translational modifications and alterative splicing (Pasciuto and Bagni, 2014a; Bonaccorso et al., 2015). For instance, the *Drosophila* dNab2 protein (the ortholog of the human ZC3H14), is a polyadenosin RNA-binding protein that has been related to autosomal recessive intellectual disability (Pak et al., 2011). In the olfactory system of the fly, MB axons lacking dNab2 exhibit disrupted development, projecting aberrantly across the brain midline and show defective branching, which leads to short-term memory impairment (Kelly et al., 2016).

Interestingly, dNab2 interacts with dFMRP in cultured brain neurons and co-distribute in the different neuronal compartments. In the olfactory system, dNab2 and dFMRP are strongly expressed in the PN, where dNab2 can interact with and regulate CaMKII mRNA, a dFMRP target. Furthermore, flies carrying mutations in both *dNab2* and *dfmrp* genes show an impaired aversive-odor induced suppression of phototaxis indicating that the dNab2 and dFMRP interaction are required for olfactory memory (Bienkowski et al., 2017). Another protein that interacts with dFMRP is Ataxin-2 (Atx-2). Atx-2 is an RNA-binding protein related to neurodegeneration since mutations resulting in the expansion of a polyglutamine tract in the gene encoding ataxin-2 give rise to the neurodegenerative disorders spinocerebellar ataxia type 2 and Parkinson’s disease (Satterfield and Pallanck, 2006). It has been shown that dFMRP and Atx-2 proteins physically associates and bind with the *CaMKII* mRNA. In this context, it has been suggested that dFMR1 is required for long-term olfactory habituation (LTH), a phenomenon dependent on Atx2-dependent potentiation of inhibitory transmission from LNs to PNs in the AL (Sudhakaran et al., 2014). Dff related protein-2 (Drep-2), another protein regulating FMRP function, was first described as an apoptosis regulator in fly (Park and Park, 2012). However, it was later determined that Drep-2 expression is highly enriched at post-synaptic densities of MB input synapses in flies, where it plays a role in normal olfactory short- and intermediate-term memory, by forming a protein complex with FMRP (Andlauer et al., 2014). Interestingly, Drep-2 ablation functionally compensates for the loss a FMRP, following a proposed mechanism where Drep-2 is required downstream of mGluR signaling to counteract the translational repression executed by FMRP (Andlauer et al., 2014), thereby recapitulating the rescued phenotype of mutants lacking FMRP induced by pharmacological inhibition of mGluRs (McBride et al., 2005).

Taking together this evidence suggests that the detrimental olfactory sensing observed in FXS models may be partially explained by the altered interaction between FMRP and RNA-binding proteins, such as dNab2 and Atx-2 and other interactors such as Drep-2.

Furthermore, other molecular modifications such as post-translational modifications and alternative splicing also regulate FMRP function in the olfactory system. Of note, FMRP is phosphorylated on a specific serine residue (human S500; murine S499; *Drosophila* S406), and such phosphorylation influences the translational state of FMRP-associated polysomes (Ceman et al., 2003; Coffee et al., 2012). It has been shown that in murine models FMRP regulates post-synaptic physiology in a mechanism where activation of metabotropic glutamate receptors (mGluR) induces a rapid FMRP dephosphorylation, mediated by PP2A (Nalavadi et al., 2012) promoting then a burst in translation FMRP-bound mRNAs including the one encoding for post-synaptic density-95 protein (PSD-95; Narayanan et al., 2007; Muddashetty et al., 2011). The ectopic expression of human FMRP into a *Drosophila* model for FXS fully rescues the molecular and cellular defects at NMJ, demonstrating functional conservation among species (Coffee et al., 2010). To analyze the effect of the FMRP phosphorylation state on neuronal physiology, human FMRP dephosphomimetic (S500A-*hFMR1)* and phosphomimic (*S500D-hFMR1)* transgenes were transformed into an FXS *Drosophila* model. Interestingly, only S500D-*hFMR1* restore normal synaptic architecture in *dfmr1* null neurons and successfully rescues learning performance back to wild-type levels in a Pavlovian olfactory learning assay, while the dephosphomimetic transgene is unable to rescue learning deficits observed in the FXS model. These results demonstrate that the phosphorylation at S500 residue within human FMRP is necessary for proper olfactive sensory learning (Coffee et al., 2012).

Finally, the FMR1 gene undergoes extensive alternative splicing, and several FMR1 mRNA and FMRP isoforms have been observed in both human and mouse brain tissue. The distribution and abundance of these isoforms may be associated to differential expression and functional properties of FMRP (Pretto et al., 2015). It has been suggested that the FMRP pre-mRNA can be alternatively spliced into as many as 20 different mature transcript isoforms (Brackett et al., 2013). The expression of 12 isoforms was analyzed in different mice brain regions (isoforms 1–6 containing exon 12, and isoforms 7–12 lacking exon 12). Of note, levels of isoforms 1–6 are the highest in the hippocampus and OB. Considering that exon 12 encodes for an extended loop in the RNA-binding KH2 domain of the FRMP protein, the presence of this protein motif in the isoforms preferentially expressed in the OB may define some specific subset of RNA molecules bound to FMRP in the region involved in olfactory perception in mice (Brackett et al., 2013) and explain the wide array of anatomical alterations and odor-mediated behavioral deficits found in FXS models. The FMRP interactors studied in the fly olfactory system are summarized in Table 1. Up to date, no molecular FMRP interactors have been described in the olfactory system of rodents, however, a list of proteins and mRNA targets in other rodent brain regions have been reviewed in Davis and Broadie (2017); Suhl and Hoeffer (2017); Bagni and Zukin (2019) and nicely depicted in Pasciuto and Bagni (2014a,b).

**Table 1:** Summary of dFMRP proteins and mRNAs interactors on the fly olfactory system.

Interactor partner	Interactor function	Relevance in olfactory function	Reference
**Protein**
dNab2	RNA-binding protein	Long-term olfactoy habitutation	Bienkowski et al. (2017)
Ataxin-2	RNA-binding protein	Learning and olfactory memory	Sudhakaran et al. (2014)
Dff related protein-2 (Derp-2)	Synaptic regulator	Olfactory short- and intermediate-term memory	Andlauer et al. (2014)
**mRNA**
*Futsch (*mammal ortholog: *MAP1B)*	Synaptic regulator/microtubule binding protein	Learning and olfactory memory	Zhang et al. (2001)
*Shrub (*mammal ortholog: *Chmp4)*	Endosomal sorting	Regulation of PN innervation on central brain MB	Vita and Broadie (2017)
*CaMKII* (mammal ortholog: *CaMKII*)	Synaptic regulator	Olfactory memory	Sudhakaran et al. (2014) and Bienkowski et al. (2017)

## Concluding Remarks

The *Fmr1*-KO mouse and the *dfmr1-* fly are the most widely used model organisms in the field of FXS. Both rely on olfactory information to survive, reproduce and make context-adequate decisions. Hence, in the last years the olfactory circuitry had emerged as a very interesting experimental model to study the neurobiological basis of behavioral and neuronal alterations in FXS.

FXS individuals exhibit an hyperexcitable phenotype and a high network synchronization that translate into hyperresponsiveness to stimuli (Contractor et al., 2015). Experimental models have revealed that the putative causes for this neuronal hyperexcitation are very diverse and occur at the molecular, synaptic and circuit level. It involves the excitatory and inhibitory systems producing an alteration in synaptic excitatory/inhibitory balance that ultimately translates in cognitive and behavioral impairments. In the olfactory system, the neuronal hyperexcitation is reflected in part into neurons that are broader tuned and are less selective to odors diminishing the discrimination capacity in *dfmr-* flies. Moreover, the inhibitory system in the OB and AL is greatly disturbed in mice and flies not expressing FMRP. Any or all these findings could explain the olfactory deficits and decrease learning capacity observed in FXS.

Another key symptom that appears to be greatly disturbed in mice and flies lacking FMRP is a diminished capacity for activity-dependent structural plasticity. In the olfactory system, GCs lacking FMRP that were born in the adult brain do not regulate their dendritic branch length and number of dendritic spines accordingly with the environmental condition. The proliferation and proper integration of new-born GCs greatly enhance the plastic capacity of the olfactory systems increasing the inhibitory inputs of targeted MCs selectively playing a fundamental role in olfactory discrimination and learning. GCs in the *Fmr1*-KO mouse exhibit an unregulated increase in dendritic spines and synaptic contact reaching the same number WT mice at early time points during development. These suggest that the critical period could be reduced in FXS and that the accelerated synaptic contact formation could generate aberrant neuronal communication. Structural plasticity and the generation and loss of synaptic contacts underpin the learning ability of the brain and the rigidity observed in mice and flies without FMRP could also be central in their diminished sensory discrimination capacity.

This evidence suggests that inappropriate filtering of information is impaired in FXS, which could translate in aberrant decision-making and behavior and that disruption in the FMRP molecular interactions would explain, at least partially, the synaptic phenotype observed in the FXS models. More evidence, however, needs to be gathered *in vivo*, in freely moving animals that are actively behaving and learning to truly uncover how information processing computations are altered in FXS and advance the field to find potential therapeutic solutions.

## Author Contributions

AN-P, FB, CG-B and CC-DR reviewed the literature and wrote the manuscript. CT-M helped to review the literature.

## Conflict of Interest Statement

The authors declare that the research was conducted in the absence of any commercial or financial relationships that could be construed as a potential conflict of interest.

## References

[B1] Abrams M. T. Kaufmann W. E. Rousseau F. Oostra B. A. Wolozin B. Taylor C. V.. (1999). FMR1 gene expression in olfactory neuroblasts from two males with fragile X syndrome. Am. J. Med. Genet. 82, 25–30. 10.1002/(SICI)1096-8628(19990101)82:1<25::AID-AJMG5>3.0.CO;2-Y 9916838

[B2] Akins M. R. Leblanc H. F. Stackpole E. E. Chyung E. Fallon J. R. (2012). Systematic mapping of fragile X granules in the mouse brain reveals a potential role for pre-synaptic FMRP in sensorimotor functions. J. Comp. Neurol. 520, 3687–3706. 10.1002/cne.23123 22522693 PMC3998511

[B3] Altman J. Das G. (1965). Post-natal origin of microneurones in the rat brain. Nature 207, 953–956. 10.1038/207953a0 5886931

[B4] Andlauer T. F. M. Scholz-Kornehl S. Tian R. Kirchner M. Babikir H. A. Depner H.. (2014). Drep-2 is a novel synaptic protein important for learning and memory. Elife 3:03895. 10.7554/eLife.03895 25392983 PMC4229683

[B5] Araneda R. C. Peterlin Z. Zhang X. Chesler A. Firestein S. (2004). A pharmacological profile of the aldehyde receptor repertoire in rat olfactory epithelium. J. Physiol. 555, 743–756. 10.1113/jphysiol.2003.058040 14724183 PMC1664868

[B6] Arnett M. T. Herman D. H. McGee A. W. (2014). Deficits in tactile learning in a mouse model of fragile X syndrome. PLoS One 9:e109116. 10.1371/journal.pone.0109116 25296296 PMC4189789

[B7] Ascano M. Mukherjee N. Bandaru P. Miller J. B. Nusbaum J. D. Corcoran D. L.. (2012). FMRP targets distinct mRNA sequence elements to regulate protein expression. Nature 492, 382–386. 10.1038/nature11737 23235829 PMC3528815

[B8] Ashley C. T. Jr. Wilkinson K. D. Reines D. Warren S. T. (1993). FMR1 protein contains conserved RNP-family domains and demonstrates selective RNA binding. Science 262, 563–566. 10.1126/science.7692601 7692601

[B9] Bagni C. Zukin R. S. (2019). A synaptic perspective of fragile X syndrome and autism spectrum disorders. Neuron 101, 1070–1088. 10.1016/j.neuron.2019.02.041 30897358 PMC9628679

[B10] Bardy C. Alonso M. Bouthour W. Lledo P.-M. (2010). How, when and where new inhibitory neurons release neurotransmitters in the adult olfactory bulb. J. Neurosci. 30, 17023–17034. 10.1523/JNEUROSCI.4543-10.2010 21159972 PMC6634902

[B11] Bear M. F. Huber K. M. Warren S. T. (2004). The mGluR theory of fragile X mental retardation. Trends Neurosci. 27, 370–377. 10.1016/j.tins.2004.04.009 15219735

[B12] Bekkers J. M. Suzuki N. (2013). Neurons and circuits for odor processing in the piriform cortex. Trends Neurosci. 36, 429–438. 10.1016/j.tins.2013.04.005 23648377

[B13] Belluzzi O. Benedusi M. Ackman J. LoTurco J. J. (2003). Electrophysiological differentiation of new neurons in the olfactory bulb. J. Neurosci. 23, 10411–10418. 10.1523/jneurosci.23-32-10411.2003 14614100 PMC6741027

[B14] Bettencourt da Cruz A. Schwärzel M. Schulze S. Niyyati M. Heisenberg M. Kretzschmar D. (2005). Disruption of the MAP1B-related protein FUTSCH Leads to changes in the neuronal cytoskeleton, axonal transport defects and progressive neurodegeneration in *Drosophila*. Mol. Biol. Cell 16, 2433–2442. 10.1091/mbc.e04-11-1004 15772149 PMC1087247

[B15] Bienkowski R. S. Banerjee A. Rounds J. C. Rha J. Omotade O. F. Gross C.. (2017). The conserved, disease-associated RNA binding protein dNab2 interacts with the fragile X protein ortholog in *Drosophila* neurons. Cell Rep. 20, 1372–1384. 10.1016/j.celrep.2017.07.038 28793261 PMC5577809

[B16] Bodaleo F. J. Gonzalez-Billault C. (2016). The pre-synaptic microtubule cytoskeleton in physiological and pathological conditions: lessons from *Drosophila* fragile X syndrome and hereditary spastic paraplegias. Front. Mol. Neurosci. 9:60. 10.3389/fnmol.2016.00060 27504085 PMC4958632

[B17] Bolduc F. V. Bell K. Cox H. Broadie K. S. Tully T. (2008). Excess protein synthesis in *Drosophila* fragile X mutants impairs long-term memory. Nat. Neurosci. 11, 1143–1145. 10.1038/nn.2175 18776892 PMC3038669

[B18] Bonaccorso C. M. Spatuzza M. Di Marco B. Gloria A. Barrancotto G. Cupo A.. (2015). Fragile X mental retardation protein (FMRP) interacting proteins exhibit different expression patterns during development. Int. J. Dev. Neurosci. 42, 15–23. 10.1016/j.ijdevneu.2015.02.004 25681562

[B19] Brackett D. M. Qing F. Amieux P. S. Sellers D. L. Horner P. J. Morris D. R. (2013). FMR1 transcript isoforms: association with polyribosomes; regional and developmental expression in mouse brain. PLoS One 8:e58296. 10.1371/journal.pone.0058296 23505481 PMC3591412

[B20] Brown V. Jin P. Ceman S. Darnell J. C. O’Donnell W. T. Tenenbaum S. A.. (2001). Microarray identification of FMRP-associated brain mRNAs and altered mRNA translational profiles in fragile X syndrome. Cell 107, 477–487. 10.1016/s0092-8674(01)00568-2 11719188

[B21] Buck L. Axel R. (1991). A novel multigene family may encode odorant receptors: a molecular basis for odor recognition. Cell 65, 175–187. 10.1016/0092-8674(91)90418-x 1840504

[B22] Caroni P. Donato F. Muller D. (2012). Structural plasticity upon learning: regulation and functions. Nat. Rev. Neurosci. 13, 478–490. 10.1038/nrn3258 22714019

[B23] Cea-Del Rio C. A. Huntsman M. M. (2014). The contribution of inhibitory interneurons to circuit dysfunction in fragile X syndrome. Front. Cell. Neurosci. 8:245. 10.3389/fncel.2014.00245 25202236 PMC4142705

[B24] Ceman S. O’Donnell W. T. Reed M. Patton S. Pohl J. Warren S. T. (2003). Phosphorylation influences the translation state of FMRP-associated polyribosomes. Hum. Mol. Genet. 12, 3295–3305. 10.1093/hmg/ddg350 14570712

[B25] Christie S. B. Akins M. R. Schwob J. E. Fallon J. R. (2009). The FXG: a pre-synaptic fragile X granule expressed in a subset of developing brain circuits. J. Neurosci. 29, 1514–1524. 10.1523/JNEUROSCI.3937-08.2009 19193898 PMC2746438

[B27] Cleland T. A. Morse A. Yue E. L. Linster C. (2002). Behavioral models of odor similarity. Behav. Neurosci. 116, 222–231. 10.1037/0735-7044.116.2.222 11996308

[B28] Coffee R. L. Tessier C. R. Woodruff E. A. Broadie K. (2010). Fragile X mental retardation protein has a unique, evolutionarily conserved neuronal function not shared with FXR1P or FXR2P. Dis. Model. Mech. 3, 471–485. 10.1242/dmm.004598 20442204 PMC2898537

[B29] Coffee R. L. Williamson A. J. Adkins C. M. Gray M. C. Page T. L. Broadie K. (2012). *In vivo* neuronal function of the fragile X mental retardation protein is regulated by phosphorylation. Hum. Mol. Genet. 21, 900–915. 10.1093/hmg/ddr527 22080836 PMC3263990

[B30] Comery T. A. Harris J. B. Willems P. J. Oostra B. A. Irwin S. A. Weiler I. J.. (1997). Abnormal dendritic spines in fragile X knockout mice: maturation and pruning deficits. Proc. Natl. Acad. Sci. U S A 94, 5401–5404. 10.1073/pnas.94.10.5401 9144249 PMC24690

[B31] Contractor A. Klyachko V. A. Portera-Cailliau C. (2015). Altered neuronal and circuit excitability in fragile X syndrome. Neuron 87, 699–715. 10.1016/j.neuron.2015.06.017 26291156 PMC4545495

[B32] Darnell J. C. Klann E. (2013). The translation of translational control by FMRP: therapeutic targets for FXS. Nat. Neurosci. 16, 1530–1536. 10.1038/nn.3379 23584741 PMC3999698

[B33] Darnell J. C. Van Driesche S. J. Zhang C. Hung K. Y. S. Mele A. Fraser C. E.. (2011). FMRP stalls ribosomal translocation on mRNAs linked to synaptic function and autism. Cell 146, 247–261. 10.1016/j.cell.2011.06.013 21784246 PMC3232425

[B34] Daroles L. Gribaudo S. Doulazmi M. Scotto-Lomassese S. Dubacq C. Mandairon N.. (2016). Fragile X mental retardation protein and dendritic local translation of the α subunit of the calcium/calmodulin-dependent kinase II messenger RNA are required for the structural plasticity underlying olfactory learning. Biol. Psychiatry 80, 149–159. 10.1016/j.biopsych.2015.07.023 26372002

[B35] Davis J. K. Broadie K. (2017). Multifarious functions of the Fragile X mental retardation protein. Trends Genet. 33, 703–714. 10.1016/j.tig.2017.07.008 28826631 PMC5610095

[B36] Deng P.-Y. Rotman Z. Blundon J. A. Cho Y. Cui J. Cavalli V.. (2013). FMRP regulates neurotransmitter release and synaptic information transmission by modulating action potential duration via BK channels. Neuron 77, 696–711. 10.1016/j.neuron.2012.12.018 23439122 PMC3584349

[B37] Doll C. A. Broadie K. (2015). Activity-dependent FMRP requirements in development of the neural circuitry of learning and memory. Development 142, 1346–1356. 10.1242/dev.117127 25804740 PMC4378248

[B38] Doll C. A. Vita D. J. Broadie K. (2017). Fragile X mental retardation protein requirements in activity-dependent critical period neural circuit refinement. Curr. Biol. 27, 2318–2330.e3. 10.1016/j.cub.2017.06.046 28756946 PMC5572839

[B39] Doucette W. Restrepo D. (2008). Profound context-dependent plasticity of mitral cell responses in olfactory bulb. PLoS Biol. 6:e258. 10.1371/journal.pbio.0060258 18959481 PMC2573932

[B40] Ethridge L. E. White S. P. Mosconi M. W. Wang J. Pedapati E. V. Erickson C. A.. (2017). Neural synchronization deficits linked to cortical hyper-excitability and auditory hypersensitivity in fragile X syndrome. Mol. Autism 8:22. 10.1186/s13229-017-0140-1 28596820 PMC5463459

[B41] Farbman A. (1992). Cell Biology of Olfaction. New York, NY: Cambridge University Press.

[B42] Fernández-Hernández I. Rhiner C. Moreno E. (2013). Adult neurogenesis in *Drosophila*. Cell Rep. 3, 1857–1865. 10.1016/j.celrep.2013.05.034 23791523

[B43] Franco L. M. Okray Z. Linneweber G. A. Hassan B. A. Yaksi E. (2017). Reduced lateral inhibition impairs olfactory computations and behaviors in a *Drosophila* model of fragile X syndrome. Curr. Biol. 27, 1111–1123. 10.1016/j.cub.2017.02.065 28366741 PMC5405172

[B44] Galvez R. Smith R. L. Greenough W. T. (2005). Olfactory bulb mitral cell dendritic pruning abnormalities in a mouse model of the Fragile-X mental retardation syndrome: further support for FMRP’s involvement in dendritic development. Dev. Brain Res. 157, 214–216. 10.1016/j.devbrainres.2005.03.010 15878626

[B45] Gatto C. L. Pereira D. Broadie K. (2014). GABAergic circuit dysfunction in the *Drosophila* Fragile X syndrome model. Neurobiol. Dis. 65, 142–159. 10.1016/j.nbd.2014.01.008 24423648 PMC3988906

[B46] Gheusi G. Lepousez G. Lledo P. (2012). Adult-born neurons in the olfactory bulb: integration and functional consequences. Curr. Top. Behav. Neurosci. 15, 49–72. 10.1007/7854_2012_228 22976274

[B47] Goel A. Cantu D. A. Guilfoyle J. Chaudhari G. R. Newadkar A. Todisco B. (2018). Impaired perceptual learning in a mouse model of Fragile X syndrome is mediated by parvalbumin neuron dysfunction and is reversible. Nat. Neurosci. 21, 1404–1411. 10.1038/s41593-018-0231-0 30250263 PMC6161491

[B48] Hagerman R. J. Berry-Kravis E. Hazlett H. C. Bailey D. B. Moine H. Kooy R. F.. (2017). Fragile X syndrome. Nat. Rev. Dis. Primers 3:17065. 10.1038/nrdp.2017.65 28960184

[B49] Hagerman R. J. Staley L. W. O’Connor R. Lugenbeel K. Nelson D. McLean S. D.. (1996). Learning-disabled males with a fragile X CGG expansion in the upper premutation size range. Pediatrics 97, 122–126. 8545206

[B50] Hall S. S. Burns D. D. Lightbody A. A. Reiss A. L. (2008). Longitudinal changes in intellectual development in children with fragile X syndrome. J. Abnorm. Child Psychol. 36, 927–939. 10.1007/s10802-008-9223-y 18347972 PMC4820329

[B52] He C. X. Cantu D. A. Mantri S. S. Zeiger W. A. Goel A. Portera-Cailliau C. (2017). Tactile defensiveness and impaired adaptation of neuronal activity in the *Fmr1* knock-out mouse model of autism. J. Neurosci. 37, 6475–6487. 10.1523/JNEUROSCI.0651-17.2017 28607173 PMC5511879

[B51] He C. X. Portera-Cailliau C. (2013). The trouble with spines in fragile X syndrome: density, maturity and plasticity. Neuroscience 251, 120–128. 10.1016/j.neuroscience.2012.03.049 22522472 PMC3422423

[B53] Hinds H. L. Ashley C. T. Sutcliffe J. S. Nelson D. L. Warren S. T. Housman D. E.. (1993). Tissue specific expression of FMR-1 provides evidence for a functional role in fragile X syndrome. Nat. Genet. 3, 36–43. 10.1038/ng0193-36 8490651

[B54] Hummel T. Krukkert K. Roos J. Davis G. Klämbt C. (2000). *Drosophila* Futsch/22C10 is a MAP1B-like protein required for dendritic and axonal development. Neuron 26, 357–370. 10.1016/s0896-6273(00)81169-1 10839355

[B55] Huntsman M. M. Kooy R. F. (2017). “Chapter 10 - the GABAergic system contributions to the fragile X syndrome phenotype,” in Fragile X Syndrome; From Genetics to Targeted Treatment, eds Willemsen R. Frank Kooy R. (Amsterdam: Elsevier Inc.), 205–215. 10.1016/B978-0-12-804461-2.00010-X

[B56] Juczewski K. von Richthofen H. Bagni C. Celikel T. Fisone G. Krieger P. (2016). Somatosensory map expansion and altered processing of tactile inputs in a mouse model of fragile X syndrome. Neurobiol. Dis. 96, 201–215. 10.1016/j.nbd.2016.09.007 27616423

[B57] Kanellopoulos A. K. Semelidou O. Kotini A. G. Anezaki M. Skoulakis E. M. C. (2012). Learning and memory deficits consequent to reduction of the Fragile X mental retardation protein result from metabotropic glutamate receptor-mediated inhibition of cAMP signaling in *Drosophila*. J. Neurosci. 32, 13111–13124. 10.1523/JNEUROSCI.1347-12.2012 22993428 PMC6621471

[B58] Kelly S. M. Bienkowski R. Banerjee A. Melicharek D. J. Brewer Z. A. Marenda D. R.. (2016). The *Drosophila* ortholog of the Zc3h14 RNA binding protein acts within neurons to pattern axon projection in the developing brain. Dev. Neurobiol. 76, 93–106. 10.1002/dneu.22301 25980665 PMC4644733

[B59] Kooy R. F. Jin P. Bao H. Till S. Kind P. Willemsen R. (2017). “Chapter-7 Animal models of fragile X syndrome,” in Fragile X Syndrome From Genetics to Targeted Treatment, eds Willemsen R. Frank Kooy R. (Amsterdam: Elsevier Inc.), 123–147. 10.1016/B978-0-12-804461-2.00007-X

[B60] Korsak L. I. T. Shepard K. A. Akins M. R. (2017). Cell type-dependent axonal localization of translational regulators and mRNA in mouse peripheral olfactory neurons. J. Comp. Neurol. 525, 2202–2215. 10.1002/cne.24199 28266018 PMC5820109

[B61] Laggerbauer B. Ostareck D. Keidel E. M. Ostareck-lederer A. Fischer U. (2001). Evidence that fragile X mental retardation protein is a negative regulator of translation. Hum. Mol. Genet. 10, 329–338. 10.1093/hmg/10.4.329 11157796

[B62] Lamprecht R. LeDoux J. (2004). Structural plasticity and memory. Nat. Rev. Neurosci. 5, 45–54. 10.1038/nrn1301 14708003

[B63] Larson J. Kim D. Patel R. C. Floreani C. (2008). Olfactory discrimination learning in mice lacking the fragile X mental retardation protein. Neurobiol. Learn. Mem. 90, 90–102. 10.1016/j.nlm.2008.01.002 18289890 PMC2493566

[B64] Lin S. C. Nicolelis M. A. L. (2008). Neuronal ensemble bursting in the basal forebrain encodes salience irrespective of valence. Neuron 59, 138–149. 10.1016/j.neuron.2008.04.031 18614035 PMC2697387

[B65] Liu B. Li Y. Stackpole E. E. Novak A. Gao Y. Zhao Y.. (2018). Regulatory discrimination of mRNAs by FMRP controls mouse adult neural stem cell differentiation. Proc. Natl. Acad. Sci. U S A 115, E11397–E11405. 10.1073/pnas.1809588115 30373821 PMC6275535

[B66] Lois C. Alvarez-Buylla A. (1993). Proliferating subventricular zone cells in the adult mammalian forebrain can differentiate into neurons and glia. Proc. Natl. Acad. Sci. U S A 90, 2074–2077. 10.1073/pnas.90.5.2074 8446631 PMC46023

[B67] Lois C. Alvarez-Buylla A. (1994). Long-distance neuronal migration in the adult mammalian brain. Science 264, 1145–1148. 10.1126/science.8178174 8178174

[B68] Malun D. Brunjes P. C. (1996). Development of olfactory glomeruli: temporal and spatial interactions between olfactory receptor axons and mitral cells in opossums and rats. J. Comp. Neurol. 368, 1–16. 10.1002/(sici)1096-9861(19960422)368:1<1::AID-CNE1>3.0.CO;2-7 8725290

[B69] Maurin T. Lebrigand K. Castagnola S. Paquet A. Jarjat M. Popa A.. (2018). HITS-CLIP in various brain areas reveals new targets and new modalities of RNA binding by fragile X mental retardation protein. Nucleic Acids Res. 46, 6344–6355. 10.1093/nar/gky267 29668986 PMC6158598

[B70] Mazroui R. Hout M. Tremblay S. Filion C. Labelle Y. Khandjian E. (2002). Trapping of messenger RNA by Fragile X mental retardation protein into cytoplasmic granules induces translation repression. Hum. Mol. Genet. 11, 3007–3017. 10.1093/hmg/11.24.3007 12417522

[B71] McBride S. M. J. Choi C. H. Wang Y. Liebelt D. Braunstein E. Ferreiro D.. (2005). Pharmacological rescue of synaptic plasticity, courtship behavior and mushroom body defects in a *Drosophila* model of Fragile X syndrome. Neuron 45, 753–764. 10.1016/j.neuron.2005.01.038 15748850

[B72] Miller L. J. McIntosh D. N. McGrath J. Shyu V. Lampe M. Taylor A. K.. (1999). Electrodermal responses to sensory stimuli in individuals with fragile X syndrome: a preliminary report. Am. J. Med. Genet. 83, 268–279. 10.1002/(sici)1096-8628(19990402)83:4<268::aid-ajmg7>3.0.co;2-k 10208160

[B73] Mombaerts P. Wang F. Dulac C. Chao S. K. Nemes A. Mendelsohn M.. (1996). Visualizing an olfactory sensory map. Cell 87, 675–686. 10.1016/s0092-8674(00)81387-2 8929536

[B74] Morales J. Hiesinger P. R. Schroeder A. J. Kume K. Verstreken P. Jackson F. R.. (2002). *Drosophila* fragile X protein DFXR regulates neuronal morphology and function in the brain. Neuron 34, 961–972. 10.1016/s0896-6273(02)00731-6 12086643

[B75] Mori K. Nagao H. Yoshihara Y. (1999). The olfactory bulb: coding and processing of odor molecule information. Science 286, 711–715. 10.1126/science.286.5440.711 10531048

[B76] Muddashetty R. S. Nalavadi V. C. Gross C. Yao X. Xing L. Laur O.. (2011). Reversible inhibition of PSD-95 mRNA translation by miR-125a, FMRP phosphorylation and mGluR signaling. Mol. Cell 42, 673–688. 10.1016/j.molcel.2011.05.006 21658607 PMC3115785

[B77] Murthy V. N. (2011). Olfactory maps in the brain. Annu. Rev. Neurosci. 34, 233–258. 10.1146/annurev-neuro-061010-113738 21692659

[B78] Myrick L. K. Deng P.-Y. Hashimoto H. Oh Y. M. Cho Y. Poidevin M. J.. (2015). Independent role for pre-synaptic FMRP revealed by an FMR1 missense mutation associated with intellectual disability and seizures. Proc. Natl. Acad. Sci. U S A 112, 949–956. 10.1073/pnas.1423094112 25561520 PMC4313821

[B79] Nalavadi V. C. Muddashetty R. S. Gross C. Bassell G. J. (2012). Dephosphorylation-induced ubiquitination and degradation of FMRP in dendrites: a role in immediate early mglur-stimulated translation. J. Neurosci. 32, 2582–2587. 10.1523/JNEUROSCI.5057-11.2012 22357842 PMC3427762

[B80] Narayanan U. Nalavadi V. Nakamoto M. Pallas D. C. Ceman S. Bassell G. J.. (2007). FMRP phosphorylation reveals an immediate-early signaling pathway triggered by group I mGluR and mediated by PP2A. J. Neurosci. 27, 14349–14357. 10.1523/JNEUROSCI.2969-07.2007 18160642 PMC6673448

[B81] Nelson D. Santoro M. Warren S. (2017). “Chapter 2-Fragile X syndrome genetics,” in Fragile X Syndrome: From Genetics to Targeted Treatment, eds Willemsen R. Frank Kooy R. (Amsterdam: Elsevier Inc.), 19–39. 10.1016/B978-0-12-804461-2.00010-X

[B82] Nicoll R. A. (2017). A brief history of long-term potentiation. Neuron 93, 281–290. 10.1016/j.neuron.2016.12.015 28103477

[B84] Olmos-Serrano J. L. Paluszkiewicz S. M. Martin B. S. Kaufmann W. E. Corbin J. G. Huntsman M. M. (2010). Defective GABAergic neurotransmission and pharmacological rescue of neuronal hyperexcitability in the amygdala in a mouse model of fragile X syndrome. J. Neurosci. 30, 9929–9938. 10.1523/JNEUROSCI.1714-10.2010 20660275 PMC2948869

[B85] Olsen S. R. Wilson R. I. (2008). Lateral pre-synaptic inhibition mediates gain control in an olfactory circuit. Nature 452, 956–960. 10.1038/nature06864 18344978 PMC2824883

[B86] Pak C. Garshasbi M. Kahrizi K. Gross C. Apponi L. H. Noto J. J.. (2011). Mutation of the conserved polyadenosine RNA binding protein, ZC3H14/dNab2, impairs neural function in *Drosophila* and humans. Proc. Natl. Acad. Sci. U S A 108, 12390–12395. 10.1073/pnas.1107103108 21734151 PMC3145741

[B87] Pan L. Woodruff E. Liang P. Broadie K. (2008). Mechanistic relationships between *Drosophila* fragile X mental retardation protein and metabotropic glutamate receptor A signaling. Mol. Cell. Neurosci. 37, 747–760. 10.1016/j.mcn.2008.01.003 18280750 PMC3989938

[B88] Pan L. Zhang Y. Q. Woodruff E. Broadie K. (2004). The *Drosophila* fragile X gene negatively regulates neuronal elaboration and synaptic differentiation. Curr. Biol. 14, 1863–1870. 10.1016/j.cub.2004.09.085 15498496

[B89] Park O. K. Park H. H. (2012). Dual apoptotic DNA fragmentation system in the fly: Drep2 is a novel nuclease of which activity is inhibited by Drep3. FEBS Lett. 586, 3085–3089. 10.1016/j.febslet.2012.07.056 22850116

[B90] Pasciuto E. Bagni C. (2014a). SnapShot: FMRP interacting proteins. Cell 159, 218–218.e1. 10.1016/j.cell.2014.08.036 25259928

[B91] Pasciuto E. Bagni C. (2014b). SnapShot: FMRP mRNA targets and diseases. Cell 158, 1446–1446.e1. 10.1016/j.cell.2014.08.035 25215498

[B92] Petreanu L. Alvarez-Buylla A. (2002). Maturation and death of adult-born olfactory bulb granule neurons: role of olfaction. J. Neurosci. 22, 6106–6113. 10.1523/JNEUROSCI.22-14-06106.2002 12122071 PMC6757952

[B93] Pretto D. I. Eid J. S. Yrigollen C. M. Tang H. T. Loomis E. W. Raske C.. (2015). Differential increases of specific FMR1 mRNA isoforms in premutation carriers. J. Med. Genet. 52, 42–52. 10.1136/jmedgenet-2014-102593 25358671 PMC4394606

[B94] Richardson R. T. DeLong M. R. (1991). Electrophysiological studies of the functions of the nucleus basalis in primates. Adv. Exp. Med. Biol. 295, 233–252. 10.1007/978-1-4757-0145-6_12 1776570

[B95] Roos J. Hummel T. Ng N. Klämbt C. Davis G. W. (2000). *Drosophila* futsch regulates synaptic microtubule organization and is necessary for synaptic growth. Neuron 26, 371–382. 10.1016/s0896-6273(00)81170-8 10839356

[B96] Rotschafer S. Razak K. (2013). Altered auditory processing in a mouse model of fragile X syndrome. Brain Res. 1506, 12–24. 10.1016/j.brainres.2013.02.038 23458504

[B97] Saghatelyan A. Roux P. Migliore M. Rochefort C. Desmaisons D. Charneau P.. (2005). Activity-dependent adjustments of the inhibitory network in the olfactory bulb following early postnatal deprivation. Neuron 46, 103–116. 10.1016/j.neuron.2005.02.016 15820697

[B98] Sala C. Segal M. (2014). Dendritic spines: the locus of structural and functional plasticity. Physiol. Rev. 94, 141–188. 10.1152/physrev.00012.2013 24382885

[B99] Satterfield T. F. Pallanck L. J. (2006). Ataxin-2 and its *Drosophila* homolog, ATX2, physically assemble with polyribosomes. Hum. Mol. Genet. 15, 2523–2532. 10.1093/hmg/ddl173 16835262

[B100] Schenck A. Van de Bor V. Bardoni B. Giangrande A. (2002). Novel features of dFMR1, the *Drosophila* orthologue of the fragile X mental retardation protein. Neurobiol. Dis. 11, 53–63. 10.1006/nbdi.2002.0510 12460546

[B83] Schilit Nitenson A. Stackpole E. E. Truszkowski T. L. S. Midroit M. Fallon J. R. Bath K. G. (2015). Fragile X mental retardation protein regulates olfactory sensitivity but not odorant discrimination. Chem. Senses 40, 345–350. 10.1093/chemse/bjv019 25917509 PMC4542900

[B101] Schlief M. L. Wilson R. I. (2007). Olfactory processing and behavior downstream from highly selective receptor neurons. Nat. Neurosci. 10, 623–630. 10.1038/nn1881 17417635 PMC2838507

[B102] Schmidt O. Teis D. (2012). The ESCRT machinery. Curr. Biol. 22, R116–R120. 10.1016/j.cub.2012.01.028 22361144 PMC3314914

[B103] Scotto-Lomassese S. Nissant A. Mota T. Néant-Féry M. Oostra B. A. Greer C. A.. (2011). Fragile X mental retardation protein regulates new neuron differentiation in the adult olfactory bulb. J. Neurosci. 31, 2205–2215. 10.1523/JNEUROSCI.5514-10.2011 21307257 PMC3682409

[B104] Semaniuk U. (2015). Olfactory system in *Drosophila*. J. Vasyl Stef. Precar. Natl. Univ. 2, 85–92. 10.15330/jpnu.2.1.85-92

[B105] Sherman S. L. Hunter J. E. (2017). “Chapter 4 - Epidemiology of fragile X syndrome,” in Epidemiology of Fragile X Syndrome: From Genetics to Targeted Treatment, eds Willemsen R. Frank Kooy R. (Amsterdam: Elsevier Inc.), 57–76. 10.1016/B978-0-12-804461-2.00004-4

[B106] Sidorov M. S. Auerbach B. D. Bear M. F. (2013). Fragile X mental retardation protein and synaptic plasticity. Mol. Brain 6:15. 10.1186/1756-6606-6-15 23566911 PMC3636002

[B107] Silbering A. F. Rytz R. Grosjean Y. Abuin L. Ramdya P. Jefferis G. S. X. E.. (2011). Complementary function and integrated wiring of the evolutionarily distinct *Drosophila* olfactory subsystems. J. Neurosci. 31, 13357–13375. 10.1523/JNEUROSCI.2360-11.2011 21940430 PMC6623294

[B108] Simões A. R. Rhiner C. (2017). A cold-blooded view on adult neurogenesis. Front. Neurosci. 11:327. 10.3389/fnins.2017.00327 28642678 PMC5462949

[B109] Snyder E. M. Philpot B. D. Huber K. M. Dong X. Fallon J. R. Bear M. F. (2001). Internalization of ionotropic glutamate receptors in response to mGluR activation. Nat. Neurosci. 4, 1079–1085. 10.1038/nn746 11687813

[B1080] Su C. Y. Menuz K. Carlson J. R. (2009). Olfactory perception: receptors, cells, and circuits. Cell 139, 45–59. 10.1016/j.cell.2009.09.015 19804753 PMC2765334

[B110] Sudhakaran I. P. Hillebrand J. Dervan A. Das S. Holohan E. E. Hülsmeier J.. (2014). FMRP and Ataxin-2 function together in long-term olfactory habituation and neuronal translational control. Proc. Natl. Acad. Sci. U S A 111, E99–E108. 10.1073/pnas.1309543111 24344294 PMC3890871

[B111] Suhl J. Hoeffer C. (2017). “RNA and protein targets of FMRP,” in Fragile X Syndrome: From Genetics to Targeted Treatment, eds Willemsen R. Frank Kooy R. (Amsterdam: Elsevier Inc.), 151–171. 10.1016/B978-0-12-804461-2.00008-1

[B112] Sweeney N. T. Brenman J. E. Jan Y. N. Gao F. B. (2006). The coiled-coil protein shrub controls neuronal morphogenesis in *Drosophila*. Curr. Biol. 16, 1006–1011. 10.1016/j.cub.2006.03.067 16713958

[B113] The Dutch-Belgian Fragile X Consortium Bakker C. E. Verheij C. Willemsen R. van der Helm R. Oerlemans F.. (1994). Fmr1 knockout mice: a model to study fragile X mental retardation. Cell 78, 23–33. 10.1016/0092-8674(94)90569-x 8033209

[B114] Vita D. J. Broadie K. (2017). ESCRT-III membrane trafficking misregulation contributes to fragile X syndrome synaptic defects. Sci. Rep. 7:8683. 10.1038/s41598-017-09103-6 28819289 PMC5561180

[B115] von Trotha J. W. Egger B. Brand A. H. (2009). Cell proliferation in the *Drosophila* adult brain revealed by clonal analysis and bromodeoxyuridine labelling. Neural Dev. 4:9. 10.1186/1749-8104-4-9 19254370 PMC2662830

[B116] Yan Q. J. Rammal M. Tranfaglia M. Bauchwitz R. P. (2005). Suppression of two major Fragile X Syndrome mouse model phenotypes by the mGluR5 antagonist MPEP. Neuropharmacology 49, 1053–1066. 10.1016/j.neuropharm.2005.06.004 16054174

[B117] Zhang Y. Q. Bailey A. M. Matthies H. J. G. Renden R. B. Smith M. A. Speese S. D.. (2001). *Drosophila* fragile x-related gene regulates the MAP1B homolog Futsch to control synaptic structure and function. Cell 107, 591–603. 10.1016/s0092-8674(01)00589-x 11733059

